# Supplementary material for: I-Boost: an integrative boosting approach for predicting survival time with multiple genomics platforms
Source: Genome Biol. 2019 Mar 7;20:52. doi: 10.1186/s13059-019-1640-4 (PMC6404283; doi:10.1186/s13059-019-1640-4)
Supplement: Supplementary file 1 — Fig. S1. The prognostic value of genomics data types. (PDF 38 kb) [file 13059_2019_1640_MOESM1_ESM.pdf]

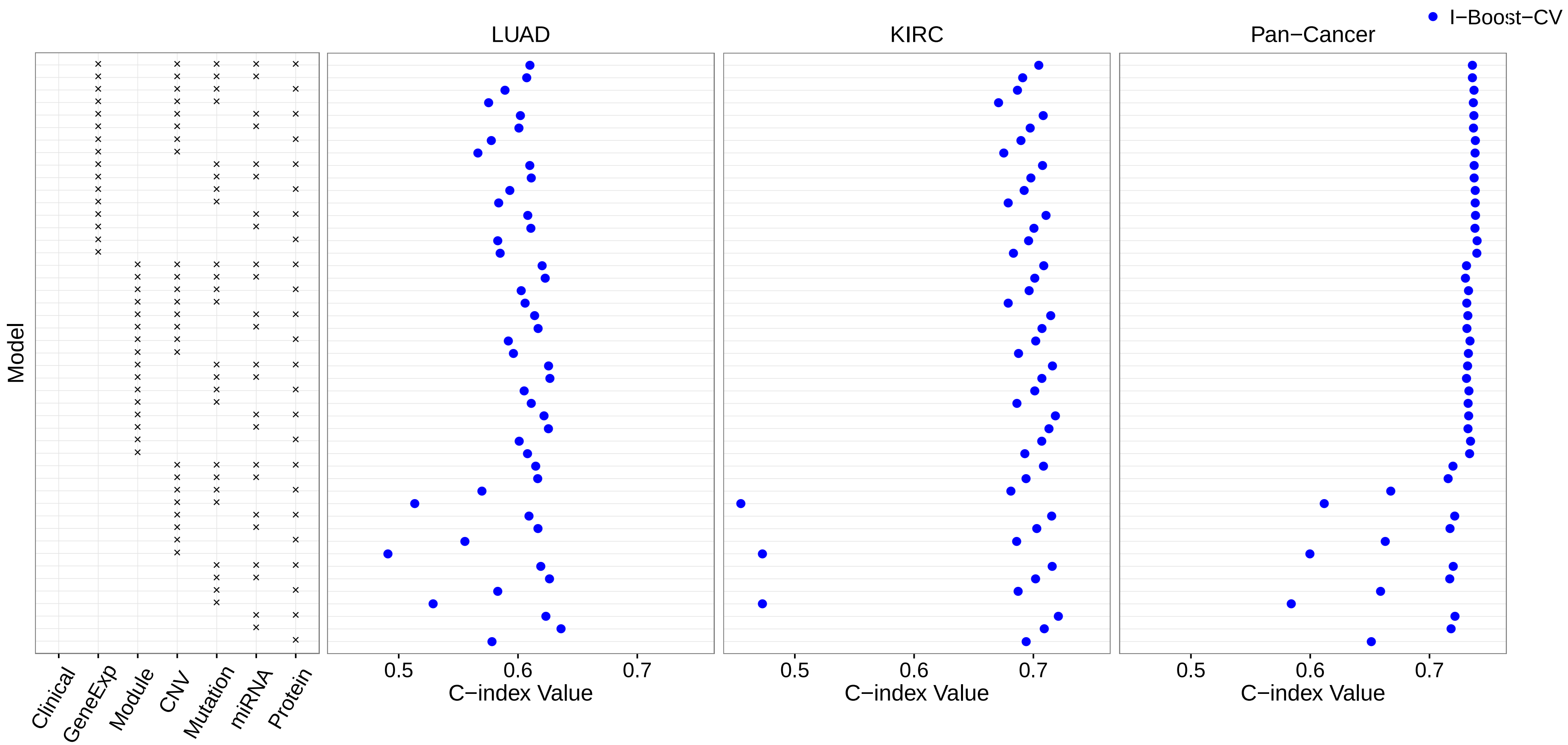

Fig. S1 Analysis results for the TCGA LUAD, KIRC, and pan-cancer data sets using I-Boost-CV. Each row represents a particular combination of data types used as predictors, as indicated by the box on the left. Each dot is an average C-index value obtained by performing I-Boost-CV on 30 training and testing data set pairs. See the caption of Fig. 1 for the abbreviations of the data types
